# Impact of varying wave periods of COVID‐19 on in‐hospital mortality and length of stay for admission through emergency department: A territory‐wide observational cohort study

**DOI:** 10.1111/irv.12919

**Published:** 2021-10-13

**Authors:** Xi Xiong, Abraham K. C. Wai, Janet Y. H. Wong, Eric H. M. Tang, Owen C. K. Chu, Carlos K. H. Wong, Timothy H. Rainer

**Affiliations:** ^1^ Centre for Safe Medication Practice and Research, Department of Pharmacology and Pharmacy, Li Ka Shing Faculty of Medicine The University of Hong Kong Hong Kong SAR China; ^2^ Emergency Medicine Unit, Li Ka Shing Faculty of Medicine The University of Hong Kong Hong Kong SAR China; ^3^ Accident and Emergency Department Queen Mary Hospital Hong Kong SAR China; ^4^ School of Nursing, Li Ka Shing Faculty of Medicine The University of Hong Kong Hong Kong SAR China; ^5^ Department of Family Medicine and Primary Care, Li Ka Shing Faculty of Medicine The University of Hong Kong Hong Kong SAR China; ^6^ Laboratory of Data Discovery for Health (D24H), Hong Kong Science and Technology Park Hong Kong SAR China

**Keywords:** cerebrovascular disease, COVID‐19, emergency admission, hospital mortality, length of stay, mental health

## Abstract

**Background:**

The COVID‐19 pandemic has been associated with excess mortality and reduced emergency department attendance. However, the effect of varying wave periods of COVID‐19 on in‐hospital mortality and length of stay (LOS) for non‐COVID disease for non‐COVID diseases remains unexplored.

**Methods:**

We examined a territory‐wide observational cohort of 563,680 emergency admissions between January 1 and November 30, 2020, and 709,583 emergency admissions during the same 2019 period in Hong Kong, China. Differences in 28‐day in‐hospital mortality risk and LOS due to COVID‐19 were evaluated.

**Results:**

The cumulative incidence of 28‐day in‐hospital mortality increased overall from 2.9% in 2019 to 3.6% in 2020 (adjusted hazard ratio [aHR] = 1.22, 95% CI 1.20 to 1.25). The aHR was higher among patients with lower respiratory tract infection (aHR: 1.30 95% CI 1.26 to 1.34), airway disease (aHR: 1.35 95% CI 1.22 to 1.49), and mental disorders (aHR: 1.26 95% CI 1.15 to 1.37). Mortality risk in the first‐ and third‐wave periods was significantly greater than that in the inter‐wave period (*p*‐interaction < 0.001). The overall average LOS in the pandemic year was significantly shorter than that in 2019 (Mean difference = −0.40 days; 95% CI −0.43 to −0.36). Patients with mental disorders and cerebrovascular disease in 2020 had a 3.91‐day and 2.78‐day shorter LOS than those in 2019, respectively.

**Conclusions:**

Increased risk of in‐hospital deaths was observed overall and by all major subgroups of disease during the pandemic period. Together with significantly reduced LOS for patients with mental disorders and cerebrovascular disease, this study shows the spillover effect of the COVID‐19 pandemic.

## 1 BACKGROUND

The coronavirus outbreak (COVID‐19) was first described in late 2019 and has brought unprecedented challenges to patients and health care systems around the world.^1^ Fear of becoming infected with COVID‐19 or a delay in seeking health care during the evolving pandemic has contributed to a significant decline in emergency hospital visits.^2^ As a result, there have been fewer emergency admissions. The decrease in emergency admissions during the early stage of the COVID‐19 pandemic has been widely reported in the United States and Europe.^3^, ^4^, ^5^, ^6^ Delays in admission and in the treatment received while in hospital may exacerbate morbidity and increase in‐hospital mortality, especially, for example, in the presence of acute cardiovascular disease.^7^, ^8^

Emergency departments (EDs) are responsible for triaging patients who need hospitalization for acute treatment or safely discharged. They also play a vital role in facilitating efficient use of resources by reducing pressure on in‐patient beds while preventing treatment delay and reducing mortality with limited resources. Before the pandemic, studies have been reported that ED crowding increased length of stay (LOS) and in‐hospital mortality.^9^ During the pandemic, the associations among ED admission, in‐hospital stay, and mortality showed changed patterns. For example, a multicenter retrospective cohort study indicated that fewer acute admissions to ED for cardiovascular emergencies but four times higher risk of death at ED and shorter LOS.^10^

Given the necessity to maximize efficient use of resources and quality of patient care in hospital, information on patterns of ED admission, LOS, and mortality,^11^ in particular, understanding the impact of COVID‐19 on these parameters over time, is critical in decision making and allocating medical resources in the most effective manner. However, there are as yet few population‐based studies comparing LOS before and during the COVID‐19 pandemic. Several studies^7^, ^10^, ^12^, ^13^ found patients with acute cardiovascular conditions in 2020 had shorter LOS, but a significant change in in‐hospital mortality in 2020 compared with 2019 has not been consistently reported.

Previous research has established that a decreased hospital admission and a higher mortality rate for non‐COVID medical conditions during the pandemic compared with the pre‐pandemic period, especially for patients admitted with respiratory diseases, cancer, pneumonia, and sepsis.^14^ However, none of the published studies has examined the in‐hospital mortality and LOS of emergency admissions. Therefore, we conducted a territory‐wide retrospective cohort study to evaluate the association of 28‐day in‐hospital mortality and LOS with emergency admission during the pre‐pandemic, pandemic wave, and interwave periods of COVID‐19 in Hong Kong, a densely populated city with prior experience of combating severe acute respiratory syndrome (SARS). Our study included all emergency admissions in January to November 2020 relative to the equivalent period in 2019. We aimed to compare the differences in a set of characteristics associated with in‐hospital mortality and LOS before and during the COVID‐19 pandemic waves. Subgroup analyses were done by patients with different medical conditions. A better understanding of the spillover effects of the COVID‐19 pandemic across waves would provide meaningful implications for healthcare leaders to help at‐risk populations during pandemic.

## 2 METHODS

### 2.1 Data source

This retrospective study was based on entries in the territory‐wide electronic health care database, the Clinical Data Analysis and Reporting System (CDARS) of the Hospital Authority, Hong Kong SAR, China. The Hong Kong Hospital Authority managed all public hospitals and mobile clinics in Hong Kong. These public hospitals with EDs supplying a population exceeded 2 million in 2019,^15^ which cover all geographical areas and provide 24‐h acute care services. Most large population‐based studies used CDARS data, and description of CDARS were well established previously.^16^, ^17^, ^18^ Extensive research has shown highly coding accuracy in CDARS.^16^, ^17^, ^18^ This study analyzed territory‐wide retrospective cohort data from all 18 EDs through CDARS, ensuring its representativeness.

### 2.2 Study population

All patients admitted as emergencies in Hong Kong between January 1, 2019, and November 30, 2019, and between January 1, 2020, and November 30, 2020, were included for analysis. We excluded hospital episodes with missing data for LOS, which represented a limited proportion of total ED admissions (0.4%). Patients who visited EDs (EDs visit) and were then admitted to hospitals were regarded as ED admissions and were included in our study as identified by records of destination of discharged at EDs. Patients who were hospitalized in public hospitals, whether transferred or directly admitted to other departments of that hospital from EDs, were included.

Our study included a list of patients' covariates, comprising age, gender, race (Chinese and non‐Chinese), living regions (Hong Kong Island, Kowloon, and New Territories), living in a residential care home for the elderly, and recipients of comprehensive social security assistance (CSSA). Severity of cases was defined by the mode of arrival, time of attendance (shift: night; day; evening), triage category, total time spent in ED (≤4 h or not), and disease diagnosis. The International Statistical Classification of Disease and Related Health Problems, Ninth Revision (ICD‐9‐CM) diagnosis codes were used to identify primary diagnosis of the following diseases: lower respiratory tract infection (LRTI), airway disease, hypertension, coronary heart disease (CHD), cerebrovascular disease and myocardial infarction (MI), diabetes, mental disorders, sepsis, chronic kidney diseases, cancer, and COVID‐19‐related conditions. The specific diagnosis for admitted patients was recorded during hospitalization other than diagnosis from ED. A social deprivation index (SDI), an indicator of socio‐economic status, was measured for each district by averaging six related variables.^19^ A set of hospital‐related variables included hospital academic status and hospital size.

**FIGURE 1:**
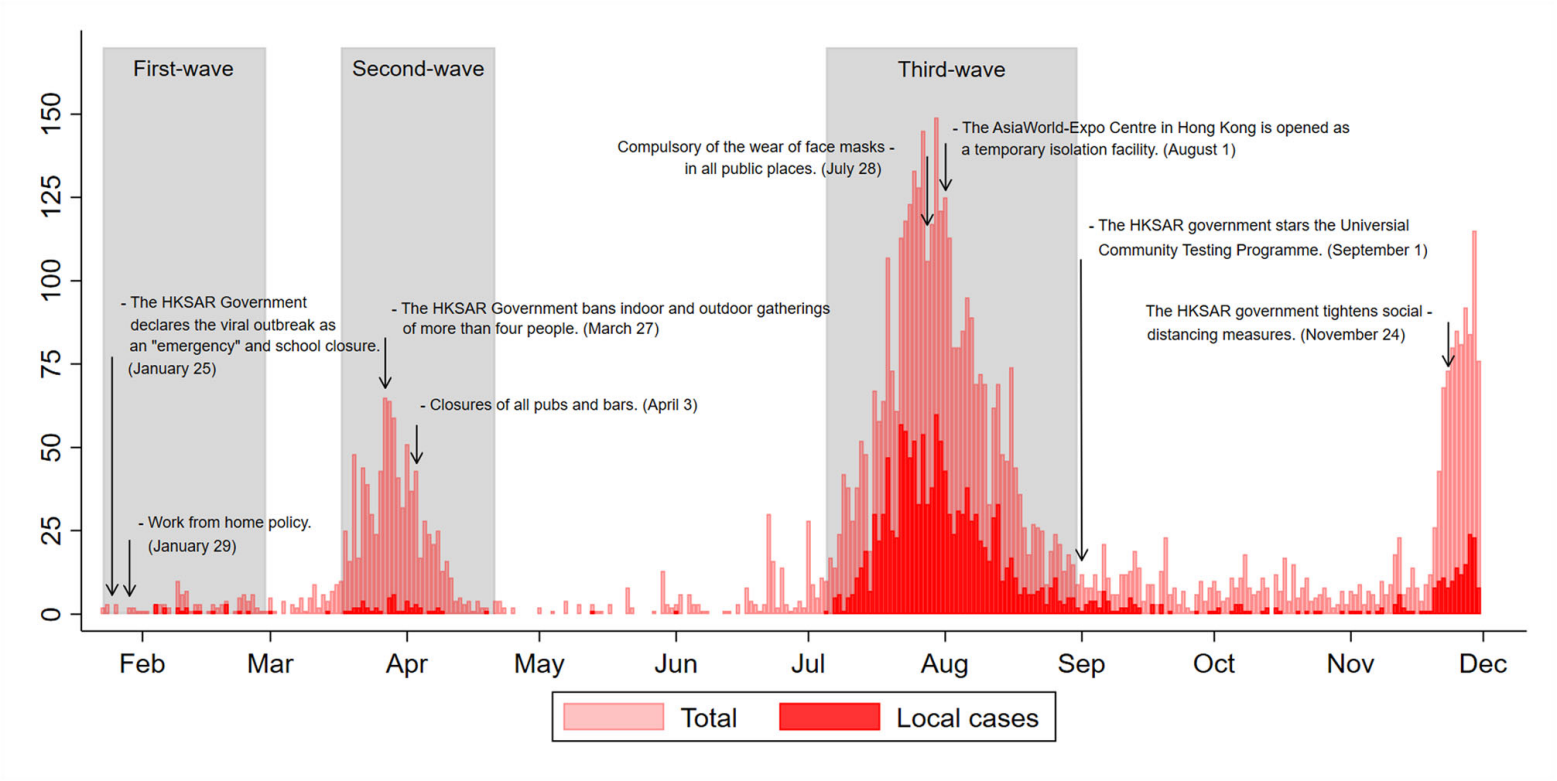
Number of COVID‐19 cases by date of reporting and timelines of interventions in Hong Kong. The epidemic curve shows the reported cases and key interventions that have occurred to date from January 1 to November 30, 2020 (gray shading areas for the three waves of COVID‐19 pandemic). The first confirmed COVID‐19 case in Hong Kong was announced on January 23, 2020: first wave from January 23 to February 29; second wave from March 17 to April 21; third wave from July 5 to August 31, 2020. *Y* axis refers to numbers of confirmed cases. The definition of local cases according to whether the patient had a travel or residence history in other regions within 14 days before diagnosis and likely exposure to pathogens in those regions; the word “regions” here refers to areas outside of Hong Kong

The first confirmed COVID‐19 case in Hong Kong was announced on January 23, 2020.^20^ We specified different wave periods in our analysis: first‐wave period from January 23 to February 29, second wave from March 17 to April 21, third wave from July 5 to August 31, and interwave period from March 1 to March 16, April 22 to July 4, and September 1 to November 30, 2020. Cohort data were extracted on February 3, 2021 (Figure 1). Cohort data were extracted on February 3, 2021. The end of each wave was defined as the local cases of the day less than 5. The beginning of the second and third waves was determined as emerge of the first local cases after the last waves and the local cases increased consecutively afterward.

### 2.3 Outcome measures

The primary outcome was the time from emergency admission to in‐hospital death within the 28‐day follow‐up period. The original definition of 28‐day in‐hospital mortality included all deaths that occurred during hospitalization within 28 days but excluding deaths that occurred after discharge from hospital. The International Statistical Classification of Diseases and Related Health Problems, Tenth Revision (ICD‐10) diagnosis codes were used to identify the primary cause of death. Diagnosis codes are listed in Table S1. The secondary outcome of this study was LOS, which was counted as the number of complete or partial days spent in hospital after attendance at an ED.

### 2.4 Data analysis

Descriptive statistics of patients' covariates including demographic and clinical characteristics, and the LOS of each hospital episode in 2019 and 2020 were displayed separately. Balance of patients' covariates between the 2 years was evaluated using the absolute standardized mean difference (SMD). An SMD <0.1 implied a covariate balance between the years.^21^

The incidence rate was a more precise estimate of the true risk of death in the dynamic populations.^22^ The weekly in‐hospital mortality rate was calculated by using the weekly number of in‐hospital deaths divided by person‐days at risk of each week during the period of hospitalization or 28 days, whichever was the earlier. Besides, to show the daily risk of death after admission by years, incidence rates from Day 0 to Day 28 were calculated.

In order to examine the heterogeneous effects of COVID‐19 on in‐hospital mortality across the years and waves, the primary end point of time from emergency admission to in‐hospital death before and after pandemic or during different pandemic waves in 2020 was assessed by a Kaplan–Meier plot and compared with a log‐rank test. Cox proportional hazard models were performed to assess the impact of COVID‐19 on incidence of in‐hospital death within 28 days. Each hospital episode was observed from the date of emergency admission to one of the following events: discharge or 28 days as right censoring. Hazard ratio (HR) with 95% confidence interval (CI) for 2020 versus 2019 adjusted by individual and hospital characteristics was reported for the overall study population and the different subgroups. Proportional hazard assumptions were tested by using a log–log plot for categorical variables. There was no evidence to reject the proportional hazards assumption.

For the secondary outcome measurements, the mean differences in LOS between the 2 years were generated by linear regression after adjusting for individual and hospital characteristics. Patients who died on the day of hospital admission would have a follow‐up time and an LOS of 0.5 days for consistency with another study,^23^ assuming that death occurred in the middle of the day that death was registered.

A *p* value < 0.05 was regarded as statistically significant. All data management and statistical analyses were performed by using STATA version 16.0 (College Station, TX: StataCorp LLC).

## 3 RESULTS

A total of 1,273,263 emergency admissions were included during the 2‐year study period. Compared with 2019 (*n* = 709,583), there was a 20.6% reduction in hospital admissions in 2020 (*n* = 563,680); 2.9% (*n* = 20,519) and 3.6% (*n* = 20,502) of these patients eventually died within 28 days of emergency admission during hospitalization in 2019 and 2020, respectively. The mean LOS decreased slightly from 5.49 days in 2019 to 5.17 days in 2020 (*p* < 0.001).

Table 1 illustrates the demographics and clinical characteristics of the emergency admissions in the 2019 and 2020 cohorts. Characteristics of hospital episodes by years were similar (all SMDs < 0.1). Of 563,680 emergency admissions in 2020, 2111 (0.4%) were admitted due to COVID‐19 diagnosis or treatment, and 1194 (0.2%) were screened for COVID‐19 during hospitalization.

**TABLE 1:** Demographics, clinical characteristics of emergency admissions on January 1–November 30, 2019, and January 1–November 30, 2020

*N* = 1,273,263	2019 (*N* = 709,583)	2020 (*N* = 563,680)	SMD
	*N* (%)	*N* (%)	
Aged over 65	366,784 (51.7)	299,701 (53.2)	0.030
Male	349,241 (49.2)	282,065 (50.0)	0.016
Chinese	655,583 (92.4)	519,146 (92.1)	0.011
Region			0.018
Hong Kong	115,249 (16.2)	91,927 (16.3)	‐
Kowloon	217,647 (30.7)	177,339 (31.5)	‐
New Territories	376,687 (53.1)	294,414 (52.2)	‐
Ambulance	358,523 (50.5)	298,912 (53.0)	0.050
Residential care home	86,893 (12.2)	75,960 (13.5)	0.037
CSSA	249,587 (35.2)	203,960 (36.2)	0.021
SDI			0.013
Low	90,236 (12.7)	70,433 (12.5)	‐
Middle	516,212 (72.7)	408,997 (72.6)	‐
High	103,135 (14.5)	84,250 (14.9)	‐
Pre‐pandemic	49,578 (7.0)	45,602 (8.1)	0.042
Waves			0.073
Wave 1	77,828 (11.0)	55,711 (9.9)	‐
Wave 2	79,415 (11.2)	54,699 (9.7)	‐
Wave 3	119,889 (16.9)	89,910 (16.0)	‐
Interwave	382,873 (54.0)	317,758 (56.4)	0.049
Triage category			0.033
Critical	13,830 (1.9)	12,287 (2.2)	‐
Emergency	44,076 (6.2)	38,855 (6.9)	‐
Urgent	461,944 (65.1)	363,530 (64.5)	‐
Semiurgent & nonurgent	189,733 (26.7)	149,008 (26.4)	‐
Shift			0.021
Night (0 am–8 am)	108,053 (15.2)	82,336 (14.6)	‐
Day (8 am–4 pm)	330,141 (46.5)	267,417 (47.4)	‐
Evening (4 pm–0 am)	271,389 (38.2)	213,927 (38.0)	‐
Total time in ED ≤ 4 h	452,010 (63.7)	363,374 (64.5)	0.016
Hospital size			0.024
Small	195,067 (27.5)	151,092 (26.8)	‐
Medium	184,648 (26.0)	152,204 (27.0)	‐
Large	329,868 (46.5)	260,384 (46.2)	‐
Teaching hospital	276,206 (38.9)	220,475 (39.1)	0.004
Disease diagnosis			
Respiratory conditions			
Lower respiratory tract infection	74,470 (10.5)	51,481 (9.1)	0.046
Airway disease	27,838 (3.9)	15,745 (2.8)	0.063
Hypertension	60,065 (8.5)	48,084 (8.5)	0.002
Coronary heart disease	24,038 (3.4)	18,766 (3.3)	0.003
Cerebrovascular disease	19,184 (2.7)	17,613 (3.1)	0.025
Myocardial infarction	9018 (1.3)	7626 (1.4)	0.007
Diabetes	37,916 (5.3)	31,165 (5.5)	0.008
Mental disorders	24,492 (3.5)	21,137 (3.7)	0.016
Sepsis	23,314 (3.3)	19,963 (3.5)	0.014
Chronic kidney diseases	15,317 (2.2)	13,461 (2.4)	0.015
Cancer	10,179 (1.4)	9010 (1.6)	0.013
COVID‐19 related			
Confirmed cases	‐	2111 (0.4)	‐
Screening for COVID‐19	‐	1194 (0.2)	‐

*Note*: The absolute SMD < 0.1 indicates covariate balance.

Abbreviations: CSSA, comprehensive social security assistance; ED, emergency department; SDI, social deprivation index; SMD, absolute standardized mean difference.

FIGURE 1 The epidemic curve shows the reported cases and key interventions that have occurred to date from January 1 to November 30, 2020 (gray shading areas for the three waves of COVID‐19 pandemic)

**FIGURE 2:**
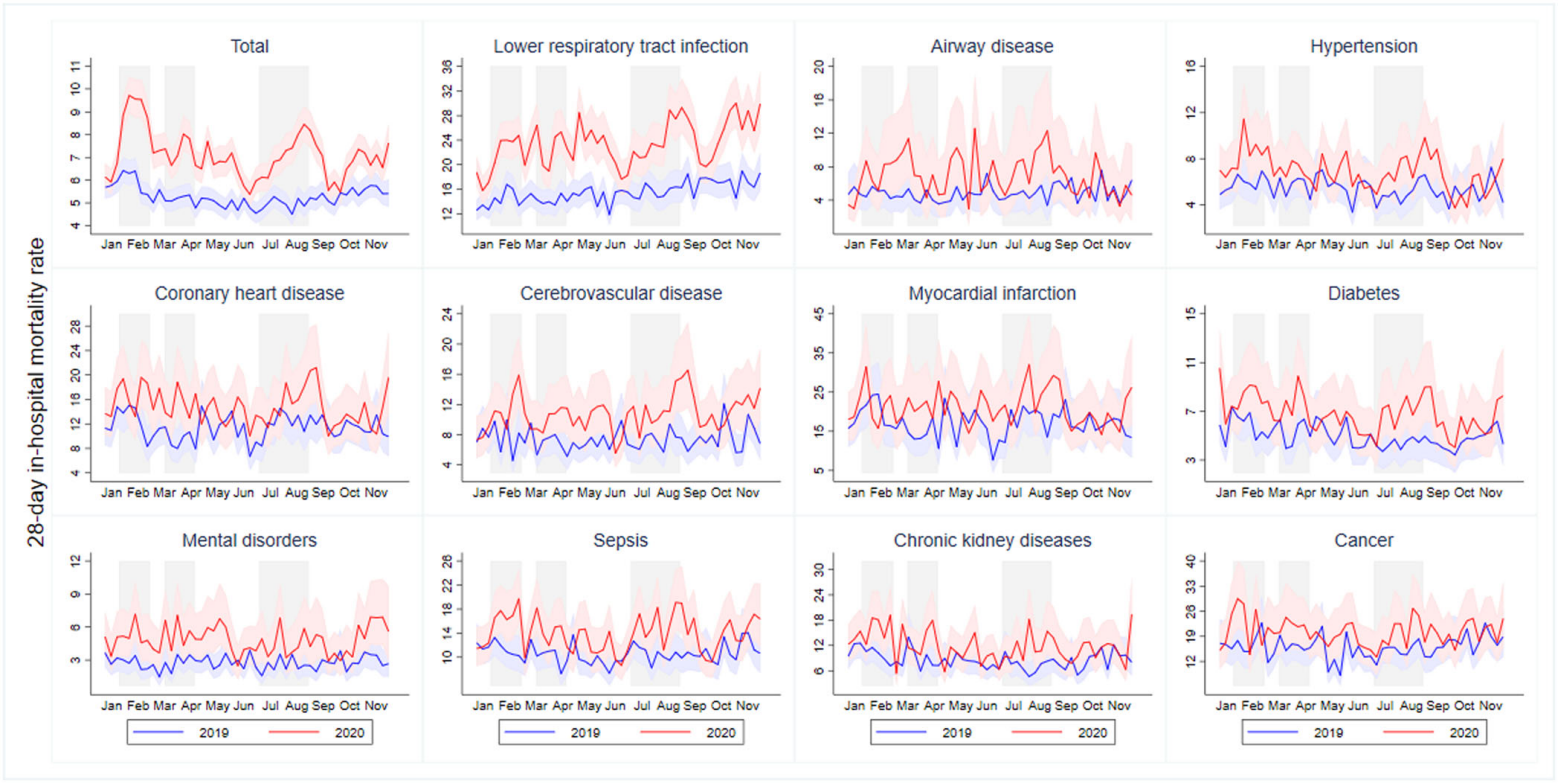
Dynamic change in weekly risk of 28‐day in‐hospital death by diagnoses. The figure shows the actual weekly risk of 28‐day in‐hospital death by diagnoses from January 1 to November 30, 2020 (red line) and the same period in 2019 (blue line). 95% confidence intervals were calculated for weekly in‐hospital mortality rate by using a Poisson distribution (red shading area for 2019 and blue shading area for 2020). Three waves were shown in gray shading

Figure 2 shows the actual weekly risk of 28‐day in‐hospital death by diagnoses for each year. The total mortality rate differed significantly by weeks, being highest in the first wave (IR of Week 5: 9.72 [95% CI 8.94 to 10.54] vs. 6.30 [95% CI 5.78 to 6.87] per 1000 person‐days in 2020 and 2019). The total mortality risk by weeks had not returned to the base level at the end of the first wave before rising to a new peak in the second wave (IR of week 14: 8.03 [95% CI 7.29 to 8.83] vs. 5.29 [95% CI 4.81 to 5.80] per 1000 person‐days in 2020 and 2019). Patients admitted in 2020 displayed peaks in 28‐day in‐hospital mortality at Weeks 5 and 14 that matched the large reduction in numbers of patients at risk of death (a decrease of 20.3% and 40.4% of admissions at Weeks 5 and 14, respectively) (Table S2). By early October, a resurgence in weekly 28‐day in‐hospital mortality had begun, with the mild reduction of ED admissions. Weekly mortality rates increased substantially for conditions such as LRTI, airway disease, hypertension, CHD, cerebrovascular disease, diabetes, and mental disorders during the pandemic wave periods. For all other diagnosis groups, the widened gaps in the weekly in‐hospital mortality rate between years were observed in the early or late of the third wave.

**FIGURE 3:**
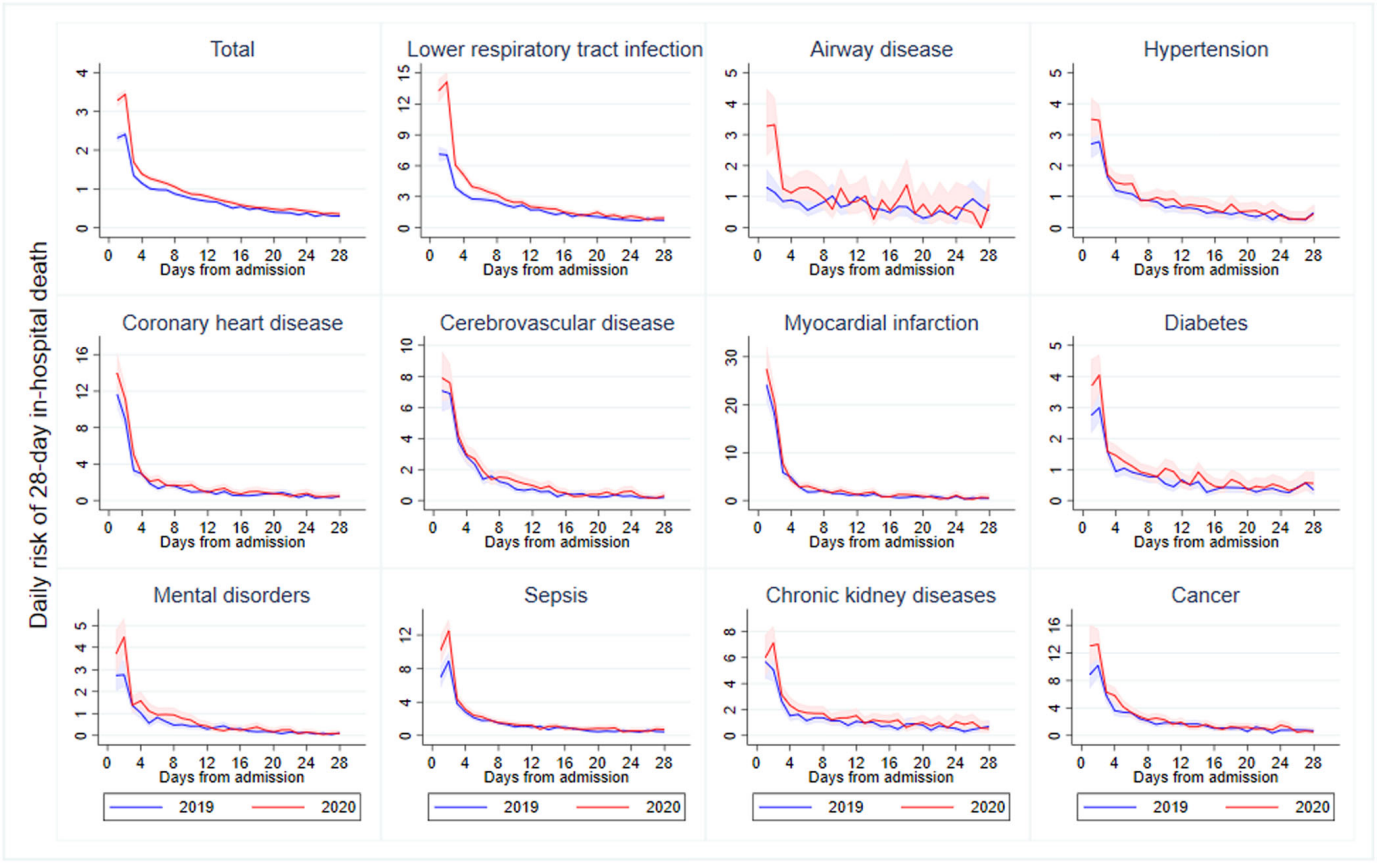
Crude daily risk of in‐hospital death from emergency admission to 28 days by diagnoses in 2019 and 2020. The figure shows the actual daily risk of 28‐day in‐hospital death from January 1 to November 30, 2020 (red line) and the same period in 2019 (blue line); 95% confidence intervals are calculated for daily in‐hospital mortality rate by using a Poisson distribution (red shading area for 2019 and blue shading area for 2020). Patients who died on the day of hospital admission would have a follow‐up time equal to 0.5 day, assuming that death occurred in the middle of the day that death was registered

Figure 3 gives the actual daily risk of 28‐day in‐hospital death by diagnoses in 2019 and 2020. Overall, the daily in‐hospital mortality in 2020 was higher than that in 2019, especially within the first 2 days (IR of the second day of admission: 3.44 [95% CI 3.33 to 3.56] vs. 2.41 [95% CI 2.32 to 2.50] per 1000 person‐days in 2020 and 2019). In both years, the daily in‐hospital mortality rate gradually decreased over the days of hospitalization. Compared with 2019, patients with LRTI, airway disease had obviously higher mortality rates on Days 1 and 2.

**FIGURE 4:**
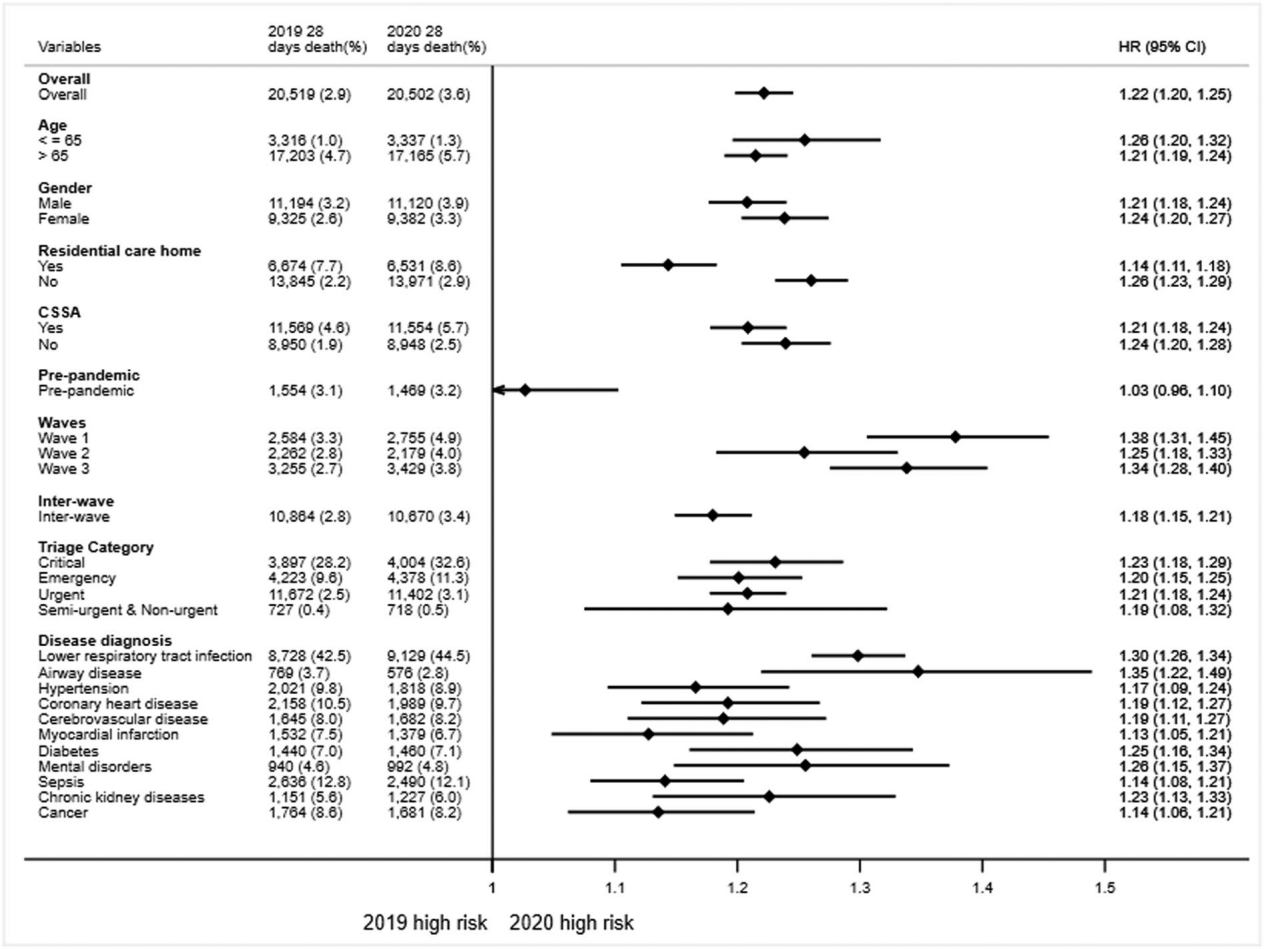
Adjusted hazard ratio (and 95% confidence interval) of 28‐day in‐hospital mortality among emergency admissions in 2020 compared with 2019. Hazard ratios were estimated using Cox proportional hazard regression models adjusting for age, gender, race, region, living in residential care home for elderly, comprehensive social security assistance (CSSA), social deprivation index (SDI), arrive mode, time of attendance, triage category, total time spend in emergency department (ED), hospital size, and academic status. The number of 28‐day in‐hospital death shows in each category. The proportion of other parameters shows the total number of deaths as proportion of hospital admissions by years. The proportion of disease diagnosis shows the number of deaths in each category as proportion of total number of deaths by years

Figure 4 compares the aHRs of 28‐day in‐hospital mortality by years. Emergency admissions in 2020 were associated with a higher risk of 28‐day in‐hospital death (aHR: 1.22 95% CI 1.20 to 1.25). The adjusted HRs for 28‐day in‐hospital mortality for all subgroups increased significantly in 2020 compared with 2019 except during the pre‐pandemic period. The differences between pandemic waves were statistically significant, according to the log‐rank test (*p* < 0.001) (Figure S1). A significantly greater effect of the COVID‐19 pandemic on in‐hospital mortality was observed in the first wave and the third wave (*p*‐interaction < 0.001) compared with the interwave period. The increase in 28‐day in‐hospital mortality in 2020 was significant, especially among patients admitted with airway disease (aHR: 1.35 95% CI 1.22 to 1.49), LRTI (aHR: 1.30 95% CI 1.26 to 1.34) and mental disorder (aHR: 1.26 95% CI 1.15 to 1.37).

**FIGURE 5:**
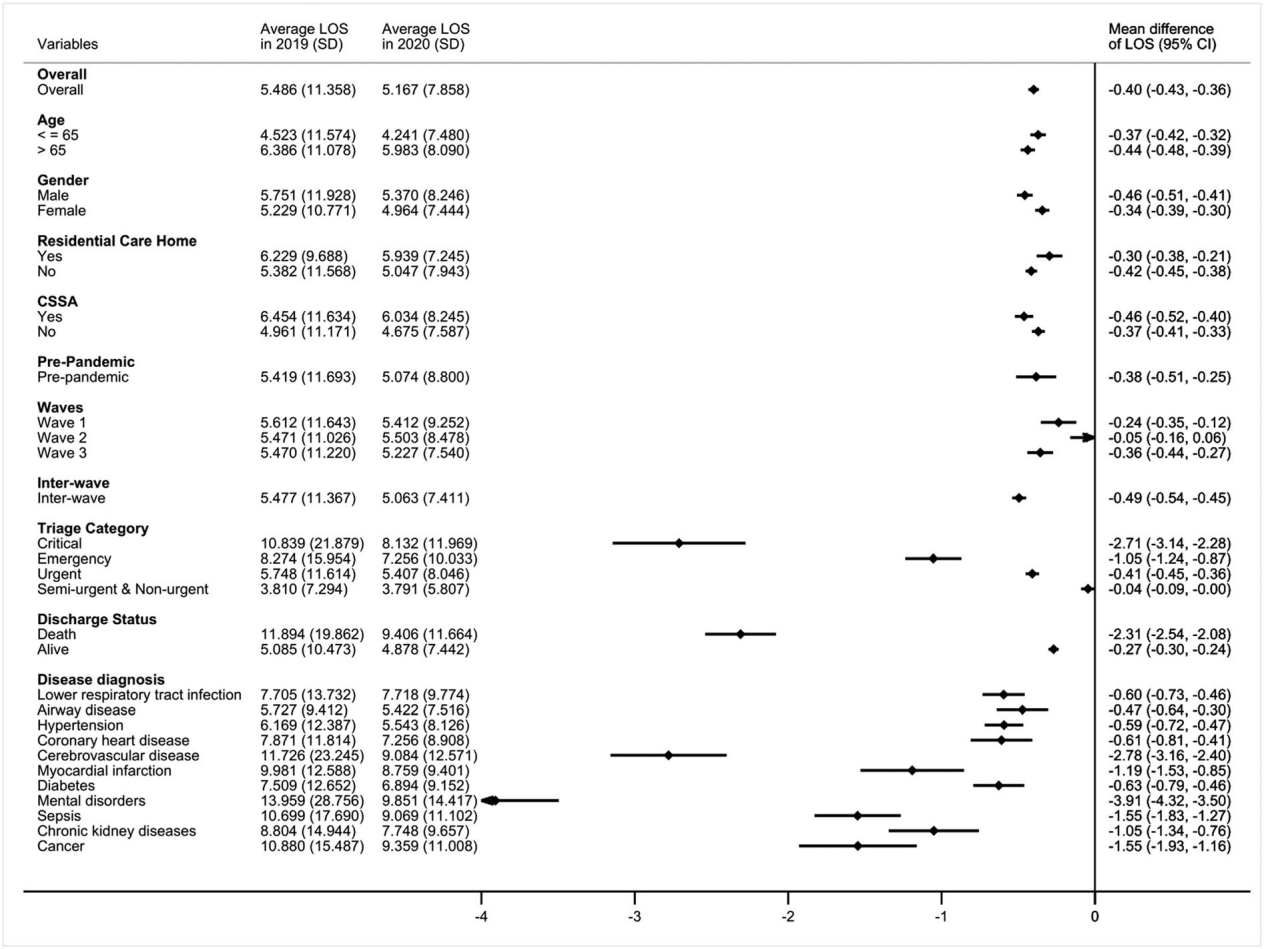
Mean difference in length of stay (LOS) (with 95% confidence interval) of emergency admissions in 2020 compared with 2019. Mean difference in LOS were estimated using linear regression models adjusting for age, gender, race, region, living in residential care home for elderly, comprehensive social security assistance (CSSA), social deprivation index (SDI), arrive mode, time of attendance, triage category, total time spend in emergency department (ED), hospital size, and academic status. Average LOS and standard deviation of each year are shown in columns

Figure 5 shows the mean differences of LOS between emergency admissions in 2020 and 2019. The mean LOS for the whole study population was 5.49 days (SD = 11.36) in 2019 and 5.17 days (SD = 7.86) in 2020. Patients triaged as critical in ED in 2020 had a 2.71‐day (95% CI 2.28 to 3.14) shorter LOS than those in 2019. The decrease in LOS was greater for those patients who died during hospitalization in 2020, whose hospital stay was on average 2.31 days (95% CI 2.08 to 2.54) shorter than patients in 2019. The decrease in LOS was largely contributable to patients admitted to hospital due to mental disorders, cerebrovascular disease, sepsis, and cancer.

## 4 DISCUSSION

### 4.1 Principal results

This population‐based cohort study reported the time course of 28‐day in‐hospital mortality and LOS among patients admitted as emergencies before and during the COVID‐19 pandemic. 20,502 (3.6%) and 20,519 (2.9%) people died 28 days within the hospital in 2020 and 2019, respectively, while a sharp decrease (20.6%) in emergency admissions during pandemic year may have contributed to a higher in‐hospital mortality rate. In 2020, significantly higher 28‐day in‐hospital mortality rates after adjusting were observed overall and across a set of major diagnosis groups such as LRTI, airway disease, and mental disorders during the pandemic period compared with 2019. Although a minor difference of LOS in overall before and after COVID‐19 pandemic years, LOS greatly decreased for some medical conditions, for example, mental disorders, cerebrovascular disease, sepsis, and cancer.

### 4.2 Comparison with prior work

The nature of the impact of COVID‐19 on in‐hospital mortality reported from previous studies has not been consistent, probably because of differences in the data source, local government intervention, study design, and statistical methodology. Plus, the degree of pressure that hospitals have come throughout the pandemic might be another major factor. Some studies found an increase in in‐hospital mortality in 2020,^7^, ^8^, ^24^, ^25^, ^26^ some reported nonsignificant differences in in‐hospital mortality,^12^, ^13^, ^26^, ^27^ and a few^7^, ^24^, ^25^, ^27^ have compared the in‐hospital mortality in pandemic waves with the interwave periods.

On one hand, finding a balance between the urgency of medical care and the perceived risk of infection with COVID‐19 results in decreased emergency admissions. Unnecessary hospitalization may have been prevented to some extent because physicians have to rethink which treatment is truly necessary at circumstance of limited medical resource during pandemic.^28^ With reductions in emergency admissions of −28.4%, −31.1%, −25.0% during the first, second, and third wave, respectively, and more information being available about COVID‐19 patient care, it was expected that the in‐hospital mortality rate would be higher. However, an increase in in‐hospital death during pandemic waves (Table S4) implies that the higher in‐hospital mortality rate was due not only to the decrease in emergency admissions but also to more critically ill patients being admitted during the pandemic.^13^ This was borne out in our study during the pandemic year with a higher proportion of patients being admitted through EDs with critical and urgent conditions. On the other hand, imposed social restrictions mean that people who live alone are less likely to be aware of mild complications. The Hong Kong government implemented a work from home policy and from January 29, 2020 (Figure 1). The advantages of early treatment are lost by any delay in seeking medical care and further increase in‐hospital mortality and the burden on hospitals.^8^

The trend towards a significant increase in in‐hospital mortality in the third wave is the more worrying. Unlike the similar increase patterns for in‐hospital mortality and confirmed COVID‐19 cases for the first wave, the in‐hospital mortality grows gradually after the beginning of the third wave in all major diagnosis groups (Figure 2). A U.S. study also showed an increase in non‐COVID‐19 mortality in countries with higher COVID‐19 mortality rates, probably resulting from weak public health infrastructure.^29^ A previous report warned that later presentation of infection will result in higher acuity of disease and, therefore, an increase in‐hospital mortality in later periods of the pandemic.^13^ Additionally, the observed higher in‐hospital mortality rate during the third wave is likely to be the result of overcrowding in the ED and a resulting increased hospital occupancy,^30^ which is reflected in our data with a higher proportion of patients spending more than 4 hours in EDs during the third wave (Table S3). As the surge in confirmed COVID‐19 cases put increased pressure on hospital capacity during the third wave,^31^ the AsiaWorld‐Expo Centre in Hong Kong was opened as a temporary isolation facility on August 1, 2020 to help cope with the increase in cases (Figure 1).^32^

Notably, our findings show an increased risk of death in patients with non‐COVID‐related respiratory disease and mental disorders in 2020. Previous studies have reported that patients with cardiovascular conditions, cancer, and respiratory disease were considered at higher risk of death during the pandemic.^7^, ^8^, ^25^, ^26^, ^33^ A nationally representative study in China also reported additional deaths from pneumonia occurred in first 3 months of COVID‐19 pandemic in Wuhan.^26^ One local study from Hong Kong highlighted the winter peak in incidence of influenza A (H1N1) from late December 2019 to late February 2020 as coinciding with the first wave as defined in our study.^34^ However, in our study, there was an increase in the number of 28‐day in‐hospital deaths from non‐COVID pneumonia during both the first and third waves (Table S4). This suggests that when patients presented to EDs with respiratory conditions during the pandemic period, the condition was more severe than in the previous year. We speculate, but this is likely due to fear of going to hospital and repeated delays to seek medical care. Meanwhile, a higher in‐hospital mortality risk of respiratory disease may result from a reduction in denominator given that very few respiratory viruses were circulating in 2020.^35^, ^36^ Patients admitted with mental disorders also had a significantly higher in‐hospital mortality rate. Mental health problems have been reported in other studies,^37^, ^38^ and a previous Hong Kong study^39^ also reported a mental health crisis after a lengthy period of social restrictions.

Our study found statistically significant decrease in LOS during pandemic year, and the slight difference of mean LOS before and after pandemic suggests this result might not be practically significant. However, the large reduction in LOS among patients who die in hospital is most marked in those who died within 2 days after admission (Figure S2). These are to be expected, mainly as a result of a higher daily in‐hospital mortality rate within the first 2 days of admission during the pandemic year (Figure 3). The largest decreases in LOS were found among critically ill patients, especially those with mental disorders, cerebrovascular disease, sepsis, and cancer. Previous studies have reported the association of acute cardiovascular disease with a lower LOS,^7^, ^12^, ^13^ whereas data for other medical conditions are sparse. It has been speculated that the suspension of elective medical care and in‐hospital surgery would reduce in‐hospital stays^6^, ^12^ and allocate hospital capacity for the treatment of COVID‐19 cases.

This study addressed two important research gaps. First, by using the Hong Kong Hospital Authority's comprehensive and reliable database, we could examine the association of in‐hospital mortality with different clinical groups. In‐hospital mortality rates following emergency admission have varied across countries and health care jurisdictions.^40^, ^41^ Our study estimated the cumulative incidence of 28‐day in‐hospital mortality to be 2.9% before the pandemic (Figure 4), in line with that in the United States (2.7%).^41^ Our study also provides clues on the prevalence of care avoidance among patients with different medical conditions by reporting the HR for death. Moreover, the pattern of COVID‐19 wave attacks was obvious. By assessing how different waves of COVID‐19 impact these outcomes, we could have a unique opportunity to evaluate the short‐term and spillover effect of COVID‐19 pandemic on emergency admissions. Our findings offer a new perspective on and important implication for targeting vulnerable patient groups and helping to make health care decisions after the epidemic subsides or before the next wave hits. Second, this study is one of the few that has compared the change of LOS over the pandemic period in patients with different medical conditions. The comprehensiveness of the study is improved by representative data from all 18 EDs in Hong Kong. Plus, seasonal variations were considered by comparing data with the same period in 2019.

### 4.3 Limitations

However, the study has limitations. First, this is an observational study that inherited the nature of residual confounding and might not deduce the causal effects of COVID‐19 pandemic in different waves on in‐hospital mortality and LOS. However, due to small differences in measured covariates between the 2 years, increased in‐hospital mortality and decreased LOS in year 2020 were likely attributable to COVID‐19. Second, laboratory parameters, comorbidities, drugs administered, operations, and procedures performed on our cohort of emergency admissions and the utilization of the negative pressure isolation rooms were not available for this analysis. We are not able to evaluate the impact of COVID‐19 on the out‐of‐hospital death and the degree of pressure on specific specialties in hospital. However, for the variables that were available, we had over 97% complete data. Third, our data only included patients admitted through EDs and represent a specific component of hospital admissions. In general, nonemergency admitted patients have mild and less complex medical conditions^42^ and have low mortality risk and nonessential LOS. Our study evaluated the impact of COVID‐19 on patients with more severe conditions needing hospitalization. Four, the comparison was conducted between 2019 and 2020, and the effect of social unrest since second half of 2019 may complicate the result. Further broadened comparison should be done with the period range over several calendar years.

## 5 CONCLUSIONS

In 2020, patients admitted as emergencies experienced a higher 28‐day in‐hospital mortality rate and shorter LOS in Hong Kong. The trend towards a significant increase in in‐hospital mortality in the third wave shows the spillover effect of COVID‐19. Increased risk of in‐hospital deaths was observed for respiratory disease and mental disorders during the pandemic period. Together with significantly reduced LOS for patients with mental disorders and cerebrovascular disease, the COVID‐19 pandemic may result in long‐term increases in mental health problems. The results of this study provide a useful reference when preparing health precaution campaigns and for forecasting healthcare resources needed during a pandemic.

## AUTHOR CONTRIBUTIONS

**Xi Xiong:** Data curation; formal analysis; investigation; methodology; validation; visualization. **Abraham K.C. Wai:** Data curation; investigation; project administration; supervision; validation. **Eric H.M. Tang:** Formal analysis; methodology; visualization. **Owen C.K. Chu:** Data curation; formal analysis. **Carlos K.H. Wong:** Conceptualization; investigation; methodology; project administration; software; supervision; validation; visualization. **Timothy H. Rainer:** Conceptualization; investigation; project administration; supervision; validation; visualization.

## PATIENT AND OTHER CONSENTS

This study was approved and a waiver of the need for patient consent allowed by the institutional review board of the University of Hong Kong/Hospital Authority West Cluster (UW 20‐112).

### PEER REVIEW

The peer review history for this article is available at https://publons.com/publon/10.1111/irv.12919.

## Supporting information


**Table S1.** Primary disease diagnosis and causes of death defined by International Classification of Diseases, Ninth Revision, Clinical Modification (ICD‐9‐CM) diagnosis codes and International Classification of Diseases and Related Health Problems, Tenth Revision (ICD‐10) diagnosis codes.
**Table S2**. Number of hospital admission at risk and the percent decreased in 2020 per week.
**Table S3.** Demographics, clinical characteristics of emergency admissions by waves among emergency admissions on 1 January – 30 November 2019 and 1 January – 30 November 2020
**Table S4.** 28‐day in‐hospital mortality overall and by causes of death by waves among emergency admissions on 1 January – 30 November 2019 and 1 January – 30 November 2020
**Figure S1**. Kaplan–Meier survival curve for cumulative in‐hospital mortality within 28 days hospitalization by years (A) and waves (B)
**Figure S2.** The distributions of LOS for patients with different discharge status by waves and years..
**Figure S3.** Log hazard ratio (and 95% confidence interval) of 28‐day in‐hospital mortality among emergency admissions in 2020 compared with 2019

## Data Availability

The data that support the findings of this study were extracted from the Hospital Authority database in Hong Kong. Restrictions apply to the availability of these data, which were used under license for this study. Data sharing is prohibited by the Hospital Authority.
