# Supplementary material for: Impact of varying wave periods of COVID‐19 on in‐hospital mortality and length of stay for admission through emergency department: A territory‐wide observational cohort study
Source: Influenza Other Respir Viruses. 2021 Oct 13;16(2):193–203. doi: 10.1111/irv.12919 (PMC8653231; doi:10.1111/irv.12919)
Supplement: Supplementary file 1 — Table S1. Primary disease diagnosis and causes of death defined by International Classification of Diseases, Ninth Revision, Clinical Modification (ICD‐9‐CM) diagnosis codes and International Classification of Diseases and Related Health Problems, Tenth Revision (ICD‐10) diagnosis codes. Table S2. Number of hospital admission at risk and the percent decreased in 2020 per week. Table S3. Demographics, clinical characteristics of emergency admissions by waves among emergency admissions on 1 January – 30 November 2019 and 1 January – 30 November 2020 Table S4. 28‐day in‐hospital mortality overall and by causes of death by waves among emergency admissions on 1 January – 30 November 2019 and 1 January – 30 November 2020 Figure S1. Kaplan–Meier survival curve for cumulative in‐hospital mortality within 28 days hospitalization by years (A) and waves (B) Figure S2. The distributions of LOS for patients with different discharge status by waves and years.. Figure S3. Log hazard ratio (and 95% confidence interval) of 28‐day in‐hospital mortality among emergency admissions in 2020 compared with 2019 [file IRV-16-193-s001.doc]

**Supplemental Table 1. Primary disease diagnosis and causes of death defined by International Classification of Diseases, Ninth Revision, Clinical Modification (ICD-9-CM) diagnosis codes and International Classification of Diseases and Related Health Problems, Tenth Revision (ICD-10) diagnosis codes.**

| **Disease diagnosis** | | **ICD-9-CM** |
| --- | --- | --- |
|  | Lower respiratory tract infection | 480.x - 488.x |
|  | Airway disease | 493.x, 494.x, 496.x |
|  | Hypertension | 401.x - 405.x |
|  | Coronary heart disease | 410.x - 414.x |
|  | Cerebrovascular disease | 430.x - 438.x |
|  | Myocardial infarction | 410* |
|  | Diabetes | 250.x |
|  | Mental disorders | 291.x - 298.x |
|  | Sepsis | 780.9, 785.59, 038.*, 040.89 |
|  | Chronic kidney diseases | 585.x |
|  | Cancer | 160.x - 163.x, 165.0, 200.x - 208.x |
| COVID-19 related | |  |
|  | Confirmed cases | 519.8x |
|  | Screening for COVID-19 | V75.9x |
| **Causes of death** | | **ICD-10** |
|  | Pneumonia | J12-J18 |
|  | Chronic lower respiratory diseases | J40–J47 |
|  | Coronary heart disease | I21-I25 |
|  | Myocardial infarction | I21-I23 |
|  | Cerebrovascular diseases | I60-I69 |
|  | Heart failure | I50, I42.0, I11.0, I13.0, I13.2 |
|  | sepsis | A40-A41 |
|  | Cancer | C00-C97 |
|  | Nephritis, nephrotic syndrome and nephrosis | N00-N07, N17-N19, N25-N27 |
|  | Disease of digestive system | K00-K93 |
|  | COVID-19 | U071, U072 |

**Supplemental Table 2. Number of hospital** admission at risk and the percent decreased in 2020 per week.

| **Wave** | **Week** | **2019** | **2020** | **% Change** |
| --- | --- | --- | --- | --- |
|  | 1 | 15,589 | 14,942 | -4.15 |
|  | 2 | 16,005 | 14,420 | -9.90 |
| **Wave 1** | 3 | 15,814 | 14,245 | -9.92 |
|  | 4 | 15,181 | 11,605 | -23.56 |
|  | 5 | 13,878 | 11,053 | -20.36 |
|  | 6 | 13,786 | 9,983 | -27.59 |
|  | 7 | 15,032 | 9,982 | -33.59 |
|  | 8 | 15,197 | 10,380 | -31.70 |
|  | 9 | 15,680 | 9,262 | -40.93 |
| **Wave 2** | 10 | 15,065 | 10,532 | -30.09 |
|  | 11 | 15,932 | 10,981 | -31.08 |
|  | 12 | 15,688 | 11,736 | -25.19 |
|  | 13 | 15,418 | 10,328 | -33.01 |
|  | 14 | 15,546 | 9,271 | -40.36 |
|  | 15 | 15,518 | 10,197 | -34.29 |
|  | 16 | 14,692 | 11,530 | -21.52 |
|  | 17 | 15,617 | 10,993 | -29.61 |
|  | 18 | 14,733 | 11,805 | -19.87 |
|  | 19 | 15,348 | 12,320 | -19.73 |
|  | 20 | 16,071 | 12,019 | -25.21 |
|  | 21 | 15,408 | 12,496 | -18.90 |
|  | 22 | 14,998 | 12,621 | -15.85 |
|  | 23 | 15,063 | 12,914 | -14.27 |
|  | 24 | 15,165 | 13,313 | -12.21 |
|  | 25 | 15,349 | 13,315 | -13.25 |
| **Wave 3** | 26 | 15,145 | 13,141 | -13.23 |
|  | 27 | 15,213 | 12,794 | -15.90 |
|  | 28 | 15,007 | 12,142 | -19.09 |
|  | 29 | 15,004 | 10,763 | -28.27 |
|  | 30 | 14,439 | 10,287 | -28.76 |
|  | 31 | 13,603 | 9,879 | -27.38 |
|  | 32 | 14,646 | 10,233 | -30.13 |
|  | 33 | 14,261 | 10,442 | -26.78 |
|  | 34 | 14,016 | 11,148 | -20.46 |
|  | 35 | 14,106 | 11,096 | -21.34 |
|  | 36 | 14,399 | 11,399 | -20.83 |
|  | 37 | 14,338 | 11,585 | -19.20 |
|  | 38 | 14,991 | 12,679 | -15.42 |
|  | 39 | 14,748 | 12,354 | -16.23 |
|  | 40 | 13,797 | 12,178 | -11.73 |
|  | 41 | 13,947 | 12,138 | -12.97 |
|  | 42 | 14,329 | 12,773 | -10.86 |
|  | 43 | 14,675 | 12,552 | -14.47 |
|  | 44 | 14,778 | 12,969 | -12.24 |
|  | 45 | 14,455 | 13,035 | -9.82 |
|  | 46 | 13,811 | 13,061 | -5.43 |
|  | 47 | 14,180 | 12,863 | -9.29 |
|  | 48 | 9,922 | 9,925 | 0.03 |

**Supplementary Table 3. Demographics, clinical characteristics of emergency admissions by waves among emergency admissions on 1 January – 30 November 2019 and 1 January – 30 November 2020**

| **N = 200,320** | | **Wave 1 (N = 55,711)** | **Wave 2 (N = 54,699)** | **Wave 3 (N = 89,910)** |
| --- | --- | --- | --- | --- |
|  |  | **N (%)** | **N (%)** | **N (%)** |
| **Aged over 65** | | 30,264 (54.3) | 28,642 (52.4) | 47,574 (52.9) |
| **Male** | | 28,552 (51.3) | 27,578 (50.4) | 45,790 (50.9) |
| **Chinese** | | 51,325 (92.1) | 50,127 (91.6) | 82,777 (92.1) |
| **Region** | |  |  |  |
|  | Hong Kong | 8,957 (16.1) | 8,923 (16.3) | 14,551 (16.2) |
|  | Kowloon | 17,978 (32.3) | 1,7491 (32.0) | 28,416 (31.6) |
|  | New Territories | 28,776 (51.7) | 28,285 (51.7) | 46,943 (52.2) |
| **Ambulance** | | 30,848 (55.4) | 29,610 (54.1) | 48,757 (54.2) |
| **Residential care home** | | 8,511 (15.3) | 7,659 (14.0) | 12,466 (13.9) |
| **CSSA** | | 20,851 (37.4) | 19,751 (36.1) | 32,778 (36.5) |
| **SDI** | |  |  |  |
|  | Low | 6,630 (11.9) | 6,867 (12.6) | 11,217 (12.5) |
|  | Middle | 40,608 (72.9) | 39,622 (72.4) | 65,062 (72.4) |
|  | High | 8,473 (15.2) | 8,210 (15.0) | 13,631 (15.2) |
| **Triage Category** | |  |  |  |
|  | Critical | 1,556 (2.8) | 1,208 (2.2) | 2,029 (2.3) |
|  | Emergency | 4,240 (7.6) | 3,933 (7.2) | 6,340 (7.1) |
|  | Urgent | 34,727 (62.3) | 34,391 (62.9) | 57,144 (63.6) |
|  | Semi-urgent & Non-urgent | 15,188 (27.3) | 15,167 (27.7) | 24,397 (27.1) |
| **Shift** | |  |  |  |
|  | Night (0am - 8am) | 8,489 (15.2) | 7,976 (14.6) | 13,120 (14.6) |
|  | Day (8am - 4pm) | 26,066 (46.8) | 25,858 (47.3) | 42,868 (47.7) |
|  | Evening (4pm - 0am) | 21,156 (38.0) | 20,865 (38.1) | 33,922 (37.7) |
| **Total Time in ED < = 4 hours** | | 40,359 (72.4) | 39,384 (72.0) | 56,575 (62.9) |
| **Hospital Size** | |  |  |  |
|  | Small | 14,958 (26.8) | 14,366 (26.3) | 24,498 (27.2) |
|  | Medium | 15,615 (28.0) | 15,085 (27.6) | 24,355 (27.1) |
|  | Large | 25,138 (45.1) | 25,248 (46.2) | 41,057 (45.7) |
| **Teaching Hospital** | | 22,238 (39.9) | 21,794 (39.8) | 34,825 (38.7) |
| **Disease diagnosis** | |  |  |  |
| **Respiratory conditions** | |  |  |  |
|  | Lower respiratory tract infection | 8,415 (15.1) | 5,206 (9.5) | 7,950 (8.8) |
|  | Airway disease | 2,169 (3.9) | 1,771 (3.2) | 2,459 (2.7) |
|  | Hypertension | 4,653 (8.4) | 4,843 (8.9) | 7,453 (8.3) |
|  | Coronary heart disease | 1,915 (3.4) | 1,880 (3.4) | 2,986 (3.3) |
|  | Cerebrovascular disease | 1,784 (3.2) | 1,858 (3.4) | 2,838 (3.2) |
|  | Myocardial infarction | 850 (1.5) | 734 (1.3) | 1,261 (1.4) |
|  | Diabetes | 3,298 (5.9) | 3,295 (6.0) | 5,072 (5.6) |
|  | Mental disorders | 2,126 (3.8) | 2,241 (4.1) | 3,408 (3.8) |
|  | Sepsis | 2,191 (3.9) | 2,017 (3.7) | 3,297 (3.7) |
|  | Chronic kidney diseases | 1,509 (2.7) | 1,462 (2.7) | 2,109 (2.3) |
|  | Cancer | 870 (1.6) | 934 (1.7) | 1,482 (1.6) |
| **COVID-19 related** | |  |  |  |
|  | Diagnosis and treatment | 38 (0.1) | 268 (0.5) | 1,469 (1.6) |
|  | Screening for COVID-19 | 50 (0.1) | 612 (1.1) | 263 (0.3) |

Note: SMD: the absolute standardized mean difference; CSSA: Comprehensive social security assistance; SDI: Social deprivation index; ED: emergency department. The absolute SMD < 0.1 indicates covariate balance.

**Supplementary Table 4. 28-day in-hospital mortality overall and by causes of death by waves among emergency admissions on 1 January – 30 November 2019 and 1 January – 30 November 2020**

|  |  | **Wave 1 N (%)** | | **Wave 2 N (%)** | | **Wave 3 N (%)** | | **Total N (%)** | |
| --- | --- | --- | --- | --- | --- | --- | --- | --- | --- |
|  |  | **2019** | **2020** | **2019** | **2020** | **2019** | **2020** | **2019** | **2020** |
| **Overall^a^** | | 2,584 (3.3) | 2,755 (4.9) | 2,262 (2.8) | 2,179 (3.9) | 3,255 (2.7) | 3,429 (3.8) | 8,101 (2.9) | 8,363 (4.2) |
| **Causes of death^b^** | |  |  |  |  |  |  |  |  |
|  | Pneumonia | 1,063 (41.1) | 1,202 (43.6) | 859 (38) | 822 (37.7) | 1,185 (36.4) | 1,369 (39.9) | 3,107 (38.4) | 3,393 (40.6) |
|  | Chronic lower respiratory diseases | 25 (1.0) | 20 (0.7) | 15 (0.7) | 10 (0.5) | 24 (0.7) | 15 (0.4) | 64 (0.8) | 45 (0.5) |
|  | Coronary heart disease | 143 (5.5) | 129 (4.7) | 108 (4.8) | 110 (5.0) | 206 (6.3) | 159 (4.6) | 457 (5.6) | 397 (4.7) |
|  | Myocardial infarction | 114 (4.4) | 88 (3.2) | 82 (3.6) | 81 (3.7) | 170 (5.2) | 121 (3.5) | 366 (4.5) | 290 (3.5) |
|  | Cerebrovascular diseases | 117 (4.5) | 114 (4.1) | 107 (4.7) | 91 (4.2) | 155 (4.8) | 109 (3.2) | 379 (4.7) | 314 (3.8) |
|  | Heart failure | 67 (2.6) | 90 (3.3) | 64 (2.8) | 73 (3.4) | 81 (2.5) | 74 (2.2) | 212 (2.6) | 237 (2.8) |
|  | sepsis | 73 (2.8) | 101 (3.7) | 77 (3.4) | 86 (3.9) | 134 (4.1) | 135 (3.9) | 284 (3.5) | 322 (3.9) |
|  | Cancer | 482 (18.7) | 418 (15.2) | 500 (22.1) | 429 (19.7) | 656 (20.2) | 625 (18.2) | 1,638 (20.2) | 1,472 (17.6) |
|  | Nephritis, nephrotic syndrome and nephrosis | 89 (3.4) | 107 (3.9) | 77 (3.4) | 80 (3.7) | 111 (3.4) | 122 (3.6) | 277 (3.4) | 309 (3.7) |
|  | Disease of digestive system | 124 (4.8) | 147 (5.3) | 108 (4.8) | 123 (5.6) | 145 (4.5) | 183 (5.3) | 377 (4.7) | 453 (5.4) |
|  | COVID-19 | - | 0 (0.0) | - | 0 (0) | - | 15 (0.4) | - | 15 (0.2) |
| **Missing value** | | 32 (1.2) | 37 (1.3) | 22 (0.9) | 20 (0.9) | 36 (1.1) | 77 (2.2) | 90 (1.1) | 134 (1.6) |
| Note: * Significant differences (P < 0.05) by chi-square test. a % shows the total number of deaths as proportion of hospital admissions by waves. b % shows the number of deaths in each category as proportion of total number of deaths by waves. | | | | | | | | | |
|
|


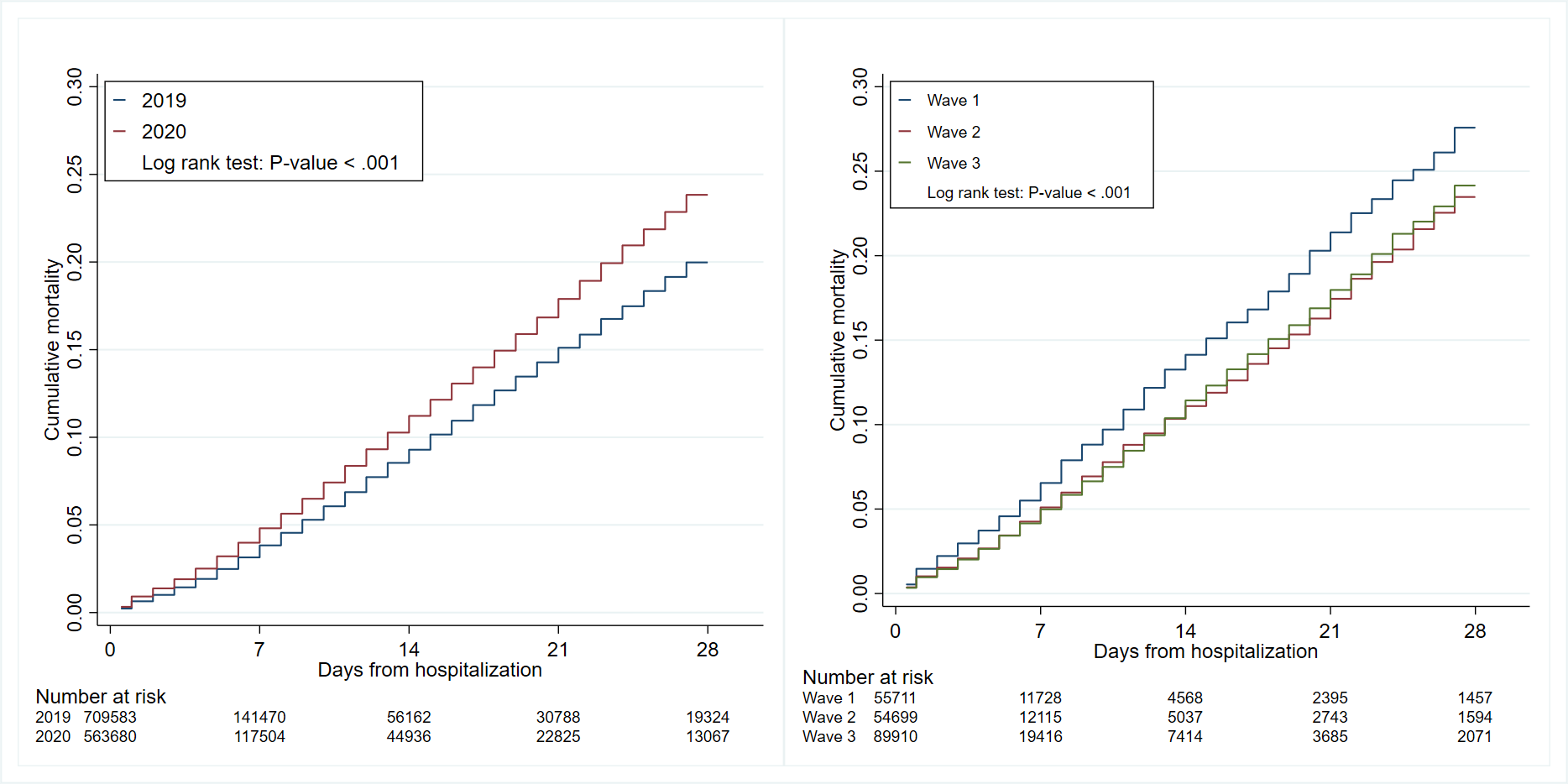


**Supplementary Figure 1. Kaplan-Meier survival curve for cumulative in-hospital mortality within 28 days hospitalization by years (A) and waves (B)**

Kaplan–Meier survival curves with number at risk shows for 28-day in-hospital mortality among emergency admissions stratified by years and waves. The difference between years and waves were statistically significant, according to log-rank test. Kaplan-Meier estimates for the number of patients at risk of death displayed as below. When stratified according to year, the patients admitted in 2020 have a higher cumulative mortality rate, indicating that there is a higher probability of death from initial date of admission (Figure 1A). To compare outcome between waves, we viewed cumulative mortality for different waves in Figure 1B and this plot showed increased mortality rate for patient admitted during the first wave. However, the curves of the second and third eaves converged over the study period.


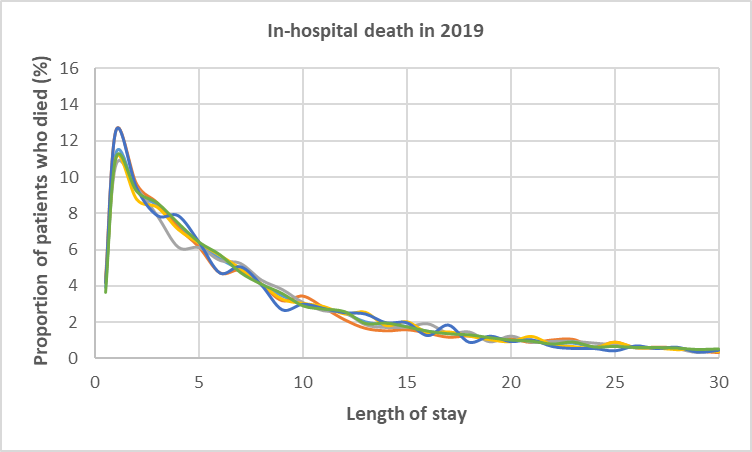

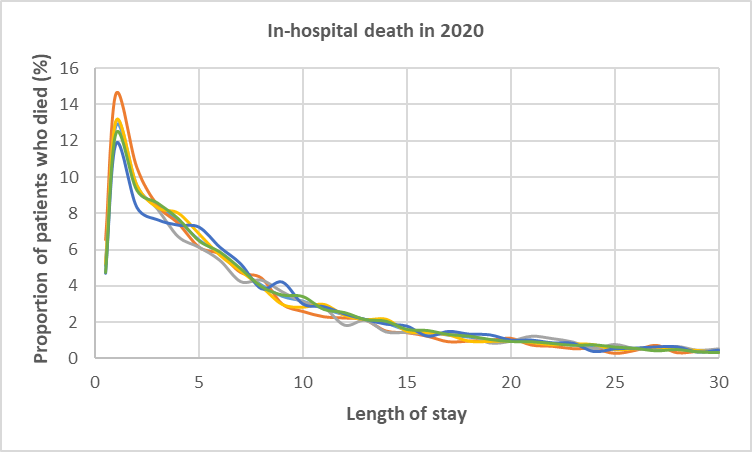


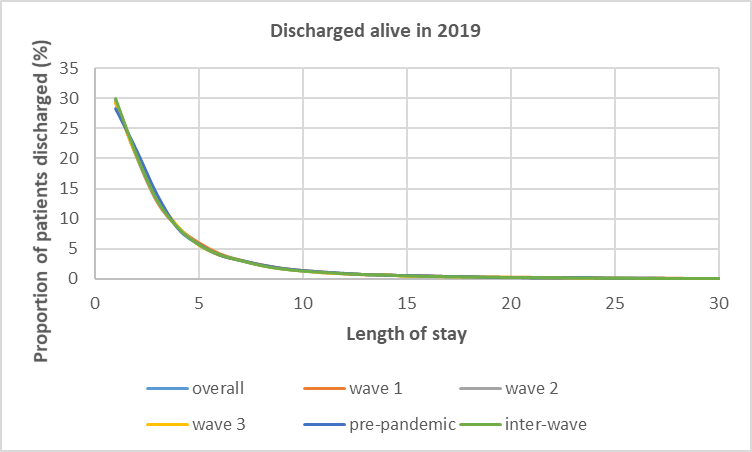

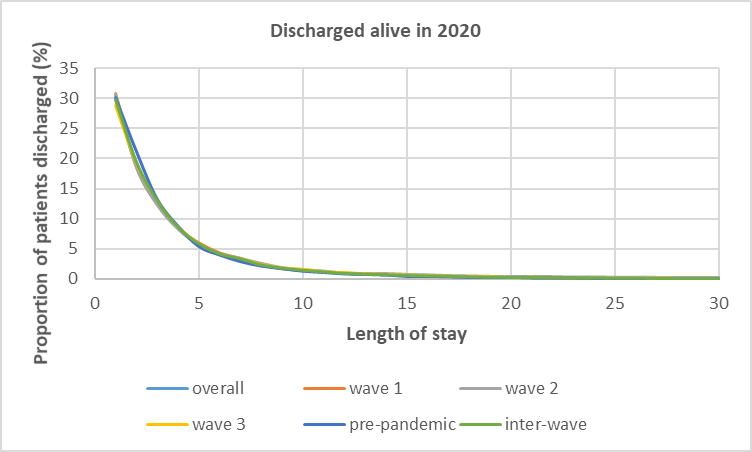


**Supplementary Figure 2. The distributions of LOS for patients with different discharge status by waves and years.**

For in-hospital death, the proportion of patients who died during hospitalization was higher at early five days in 2020 compared with 2019. Over than 27% of patients died two days after hospitalization through ED in 2020, while in 2019, accounting for less than 25% of inpatients. For patients discharged alive, patterns of distribution of LOS between years and waves almost the same.


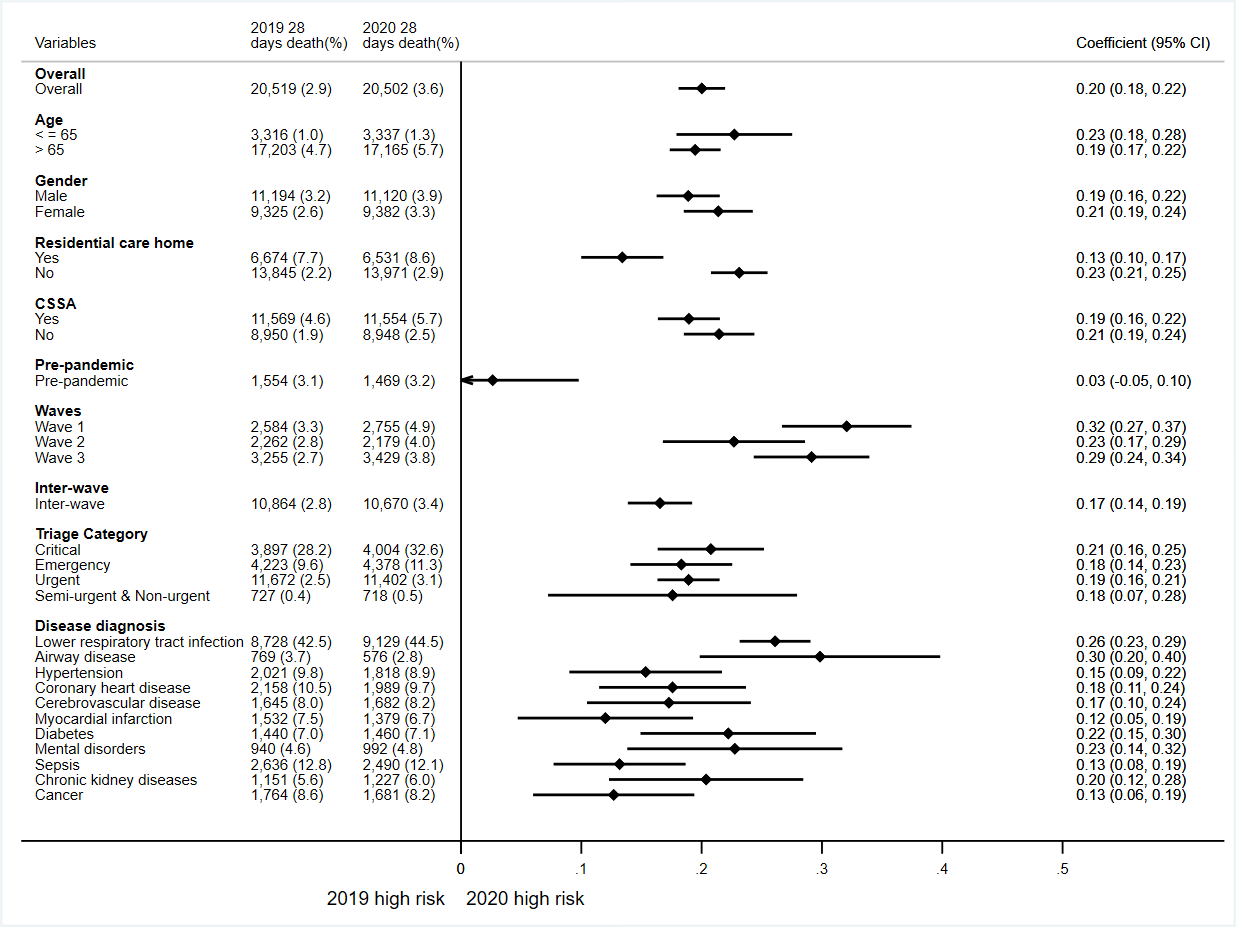


**Supplementary Figure 3. Log hazard ratio (and 95% confidence interval) of 28-day in-hospital mortality among emergency admissions in 2020 compared with 2019**
